# Acute Kidney Injury in a Case Series of Patients with Confirmed COVID-19 (Coronavirus Disease 2019): Role of Angiotensin-Converting Enzyme 2 and Renin-Angiotensin System Blockade

**DOI:** 10.1155/2020/8811931

**Published:** 2020-06-29

**Authors:** Avantika Chenna, Venu Madhav Konala, Subhasish Bose, Sasmit Roy, Bhaskar Reddy Madhira, Vijay Gayam, Srikanth Naramala, Sreedhar Adapa

**Affiliations:** ^1^Department of Internal Medicine, Division of Nephrology, Phobe Putney Memorial Hospital, Medical College of Georgia, 417 W 3^rd^ Avenue, Albany 31701, GA, USA; ^2^Department of Internal Medicine, Division of Medical Oncology, Ashland Bellefonte Cancer Center, 122 St Christopher Dr, Ashland 41169, KY, USA; ^3^Lynchburg Nephrology Physicians, Lynchburg, VA, USA; ^4^Fellow in Hematology and Oncology, Sunny Upstate Medical University, Syracuse, NY, USA; ^5^Department of Medicine, Interfaith Medical Center, Brooklyn, NY, USA; ^6^Department of Internal Medicine, Division of Rheumatology, Adventist Medical Center, Hanford 93230, CA, USA; ^7^Department of Internal Medicine, Division of Nephrology, Adventist Medical Center, Hanford 93230, CA, USA

## Abstract

The renin-angiotensin system plays a very critical role in hypertension, diabetes, and kidney and heart diseases. The blockade of the renin-angiotensin system results in the prevention of progression of renal and cardiac damage. There have been controversial hypotheses raised regarding the safety of angiotensin-converting enzyme inhibitors and angiotensin receptor blockers in COVID-19 (coronavirus disease 2019). We present the case series of four patients (2 men and 2 women; 1 Caucasian and 3 African Americans; two survived and two died) with confirmed COVID-19, presenting with respiratory symptoms and acute kidney injury, who have been on angiotensin-converting enzyme inhibitors or angiotensin receptor blockers. Membrane-bound angiotensin-converting enzyme 2 (ACE2) has been implicated as the gateway for viral entry into the human cell in causing the infection. The factors contributing to acute kidney injury are diuretics, iodinated contrast administration, hemodynamic instability apart from ACE inhibitors, and angiotensin receptor blockers. The ACE inhibitors and ARBs were stopped in these patients due to acute kidney injury. We also discussed the role of ACE2 and the renin-angiotensin system (RAS) blockade in patients with COVID-19 infection along with pathogenesis.

## 1. Introduction

The severe acute respiratory syndrome by coronavirus 2 (SARS-CoV-2) has resulted in mortality worldwide and has been declared a global pandemic. The United States has the highest number of positively tested cases in the world, and the virus has been spreading relentlessly. The lung is the main organ affected by COVID-19 resulting in respiratory failure, but there is also the involvement of other organs like the heart, kidney, and gastrointestinal tract. The patients who tend to have severe disease or need intensive care unit (ICU) admission have multiorgan involvement. Membrane-bound angiotensin-converting enzyme 2 (ACE 2) has been implicated as the gateway for viral entry into the human cell in causing the infection [1, 2].

The renin-angiotensin system (RAS) plays a very critical role in hypertension, diabetes, and kidney and heart diseases. The blockade of RAS results in the prevention of progression of renal and cardiac damage. The role of angiotensin-converting enzyme inhibitors (ACEIs) and angiotensin receptor blockers (ARBs) needs to be elucidated in COVID-19. There have been controversial hypotheses raised regarding the safety of ACEIs/ARBs in COVID-19 [1]. Here, we describe the case series of four patients with confirmed COVID-19 who developed AKI. We also discuss the role of ACE2 in pathogenesis and in AKI and the perspectives of ACEIs/ARBs in COVID-19.

### 1.1. First Case

A 49-year-old male presented to the emergency room with complaints of cough and shortness of breath started getting worse for one week. Associated symptoms included fever, chills, and generalized body aches. The patient was found to be hypoxic in the emergency room, requiring oxygen via a nasal cannula. Past medical history was significant for type 2 diabetes mellitus, hypertension, dyslipidemia, depression, and gastroesophageal reflux disease. The patient had a 28-pack-year smoking history and quit smoking four years ago. The patient denied any chest pain, orthopnea, paroxysmal nocturnal dyspnea, or swelling of his extremities. The patient denied any recent travel history. His home medications included metformin 1000 mg by mouth twice a day, hydrochlorothiazide 25 mg by mouth daily, amlodipine 10 mg by mouth daily, duloxetine 60 mg by mouth daily, atorvastatin 40 mg by mouth daily, lisinopril 40 mg by mouth daily, and aspirin 81 mg by mouth daily. The patient had no significant family history.

Initial vital signs showed a blood pressure of 132/89 mmHg, heart rate of 88 beats per minute (bpm), oxygen saturation of 80% on room air, which improved to 89% on a 5 L nasal cannula, respiratory rate of 30 breaths/min, and temperature of 99.1°F. Physical examination revealed an unkempt obese male with mild tachypnea and coarse breath sounds bilaterally. The rest of the physical examination was within normal limits.

Laboratory data revealed normal hemoglobin at 13.9 g/dL and platelet count of 220K/mm^3^. Liver function tests were within normal limits. Lactic acid was slightly elevated at 1.7 mmol/L. The rest of the laboratory data are summarized in Table 1. His influenza A and B testing was negative. Chest X-ray PA and lateral view revealed bibasilar infiltrate consistent with bilateral pneumonia. The patient had a CT of the chest with IV contrast showing bilateral ground-glass opacities. The patient's nasopharyngeal swab was sent for COVID-19 testing, and he was placed in isolation.

The patient's clinical course was complicated by transferring to the intensive care unit due to worsening hypoxic respiratory failure requiring high flow oxygen. He was subsequently intubated. The patient was started on treatment for possible community-acquired pneumonia with ceftriaxone 1 g intravenously daily and azithromycin 500 mg once followed by 250 mg by mouth daily. The patient was also started on lopinavir and ritonavir as per protocol at that time.

His clinical course was also complicated by worsening renal function with the increased blood urea nitrogen to 21 mg/dL and serum creatinine to 2.2 mg/dL. The patient's lisinopril and hydrochlorothiazide were held, and he was started on 0.9% normal saline intravenously. His urine analysis was significant for 2+ protein and no RBC casts. The patient complement levels were within normal limits. Testing for hepatitis B, hepatitis C, and HIV screen was all negative. Serum protein electrophoresis did not reveal any monoclonal protein. His COVID-19 testing returned positive. The patient's urine output subsequently continued to drop, and hence he was started on dialysis due to volume overload and hyperkalemia in the setting of acute respiratory distress syndrome type. The patient is currently receiving dialysis three times a week, and his antibiotic coverage was expanded to pulse-dose vancomycin and cefepime. The patient remains intubated and ventilated in the intensive care unit.

### 1.2. Second Case

A 48-year-old female was admitted with worsening shortness of breath for two days after attending a funeral where some people have tested positive for COVID-19. The patient complained of dry cough but denied any chest pain, orthopnea, or paroxysmal nocturnal dyspnea. The patient denied any nausea, vomiting, or diarrhea. Her past medical history was significant for hypertension, dyslipidemia, and hypothyroidism. Her surgical history was positive for thyroidectomy in 2001. The patient denied any history of smoking or illicit drug use. Her home medications included rosuvastatin 20 mg by mouth daily, hydrochlorothiazide/losartan 25–100 mg by mouth daily, amlodipine 10 mg by mouth daily, levothyroxine 150 mcg by mouth daily, and aspirin 81 mg by mouth daily.

Initial vital signs were a blood pressure of 132/56 mmHg, heart rate of 109 bpm, respiratory rate of 22 breaths/min, oxygen saturation of 80% on room air, which improved to 90% on 5 L of oxygen via a nasal cannula, and temperature of 101°F. Her physical examination was significant for morbidly obese female in mild acute distress with decreased breath sounds and positive rales bilaterally. The rest of the physical examination was unremarkable.

Laboratory testing: CBC was significant for hemoglobin of 12 g/dL and platelet count of 160K/mm^3^. Liver function testing was within normal limits. The rest of the laboratory data are summarized in Table 1. Initial arterial blood gas showed a pH of 7.3, PO_2_ of 58 mmHg, PCO_2_ of 63 mmHg, and oxygen saturation of 83% on a high flow nasal cannula at 40 liters. Influenza A and B testing was negative. Chest X-ray PA and lateral showed cardiomegaly and pulmonary edema. The patient's nasopharyngeal swab was sent for COVID-19 testing, and she was placed in appropriate isolation.

The patient was initially admitted to the medical floor, and subsequently, her oxygen requirements escalated to high flow oxygen and venti mask and she was subsequently transferred to the intensive care unit for worsening hypercapnic hypoxic respiratory failure requiring intubation and ventilation. The patient was started on intravenous Lasix 80 mg every 8 hours and was also started on hydroxychloroquine 200 mg twice a day and azithromycin 500 mg once followed by 250 mg daily for five days as per protocol. The patient's creatinine continued to increase to 3.65 mg/dL, and blood urea nitrogen increased to 92 mg/dL over three days. Lisinopril and hydrochlorothiazide were held since admission, and later Lasix was also held. Her urine analysis was negative for any protein or RBC casts. The patient is currently being monitored without any requirement for dialysis at present.

### 1.3. Third Case

A 64-year-old female was admitted with complaints of cough and shortness of breath getting worse over one week. She also had fever, nausea, vomiting, and diarrhea. The patient also complained of decreased appetite. Her cough was productive with greenish sputum. The patient denied any chest pain, orthopnea, paroxysmal nocturnal dyspnea, or pedal edema. The patient denied any history of recent travel. The patient's past medical history was significant for type 2 diabetes mellitus, hypertension, dyslipidemia, and seasonal allergies. The patient denied any history of smoking or illicit drug use. The patient's home medications included metformin 750 mg by mouth twice a day, spironolactone 25 mg by mouth daily, amlodipine 10 mg by mouth daily, and azilsartan 80 mg by mouth daily.

Initial vital signs showed a blood pressure of 132/89 mmHg, heart rate of 88 bpm, respiratory rate of 30 breaths/min, temperature of 100.1°F, and oxygen saturation of 82% on room air, which improved to 89% on 5 L of oxygen via a nasal cannula. Her physical examination revealed an obese female with mild respiratory distress. Her respiratory examination revealed coarse breath sounds bilaterally. The rest of her physical examination was unremarkable.

Laboratory testing: the patient's initial CBC revealed anemia with hemoglobin 9.5 g/dL and platelet count 121K/mm^3^. Liver function tests were within normal limits. The rest of the laboratory data are summarized in Table 1. Patient's influenza A and B testing was negative. Chest X-ray PA and lateral view suggested multifocal pneumonia. The patient had a CT angiogram of the chest with PE protocol showing bilateral ground-glass opacities concerning COVID-19. The patient's nasopharyngeal swab was sent for COVID-19 testing, and she was placed in appropriate isolation.

The patient was admitted to the medical floor initially but later transferred to the intensive care unit due to hypoxic respiratory failure requiring high flow oxygen. The patient was also started on lopinavir and ritonavir per protocol at that time. The patient's clinical course continued to get worse with continued worsening of renal function with an increase in creatinine from 1.1 mg/dL to 1.7 mg/dL and then to 6.9 mg/dL for the next 3-4 days. The patient was treated with IV fluids and intravenous bicarbonate. Her medications, azilsartan and spironolactone, were discontinued because of hypotension and worsening renal function. Urine analysis was negative for protein or RBC casts. Testing for hepatitis B and C and HIV screening were negative. Testing for antinuclear antibody, anti-neutrophilic cytoplasmic antibody, and anti-glomerular basement membrane antibodies was either negative or within normal limits. Complement levels, including C3 and C4, were 65 mg/dL and 8 mg/dL, respectively, which were low. Serum protein electrophoresis revealed no M spike.

The patient's COVID-19 testing came back as positive. Her urine output dropped to 5–10 milliliters/hour. The patient developed acute respiratory distress syndrome with fluid overload and was started on Lasix 80 mg IV every 8 hours and was also placed in a prone position. The patient failed medical management and was started on dialysis due to oliguric renal failure. Despite the above efforts, the patient's clinical condition continued to deteriorate with hypoxia and hypotension, which persisted despite being maximized on four vasopressors. The family decided to opt for comfort measures only, and the patient, unfortunately, died 11 days after the presentation.

### 1.4. Fourth Case

A 77-year-old African American male presented with a chief complaint of dry cough, shortness of breath, and fever for one week. The patient was encephalopathic on presentation and went into cardiac arrest, received 1 round of cardiopulmonary resuscitation, and was able to achieve the return of spontaneous circulation. He was then placed on the hypothermic protocol. The patient was also hypotensive, requiring norepinephrine. The patient's past medical history was significant for type 2 diabetes mellitus, hypertension, dyslipidemia, and hypothyroidism. The patient has no travel history, but the wife reported a concern that he might have encountered a person who was sick. The patient has no history of smoking or illicit drug use. His home medications were pioglitazone 30 mg by mouth daily, olmesartan 40 mg by mouth daily, levothyroxine 100 mcg by mouth daily, glimepiride 4 mg by mouth daily, fenofibrate 160 mg by mouth daily, aspirin 81 mg by mouth daily, and sitagliptin 100 mg by mouth daily. His vital signs were a blood pressure of 102/66 mmHg, heart rate of 92 bpm, respiratory rate of 26 breaths per minute, oxygen saturation of 89% on 100% FiO_2_, and temperature of 95.8°F. Physical examination revealed an obese patient who was intubated and sedated. Respiratory examination revealed coarse breath sounds bilaterally.

His laboratory examination was significant for hemoglobin 14.1 g/dL and platelet count 306K/mm^3^. The rest of the laboratory data are summarized in Table 1. Liver function tests were significant for AST elevated at 125 U/L. Lactic acid was elevated at 14 mmol/L. The patient's influenza A and B testing was negative. The patient's nasopharyngeal swab was sent for COVID-19 testing, and he was placed in isolation, with the result being positive couple of days later. A portable chest X-ray revealed bilateral pulmonary infiltrates concerning COVID-19.

All antihypertensives and hypoglycemics were held, including olmesartan. The patient was treated with aggressive IV fluid resuscitation along with IV bicarbonate. The patient was administered insulin therapy for hyperglycemia.

The patient's vasopressor requirement continued to increase due to hypotension. His renal failure continued to get worse. Dialysis was considered but was not done due to hemodynamic instability, and the patient was able to urinate. Due to persistent hypotension despite the use of vasopressors and continued hypoxia despite intubation and ventilation, after discussion with the family, the family chose to opt for do not resuscitate (DNR) and comfort care only. The patient died immediately after extubation.

## 2. Discussion

We presented the case series of four patients who were on angiotensin-converting enzyme inhibitors (ACE inhibitors) or angiotensin receptor blockers (ARBs) with COVID-19 infection and acute renal failure. We summarize the findings, including patient characteristics, comorbidities, presenting symptoms, types of ACE inhibitors, or ARB use, including diuretic use at home as well as in the hospital, whether the patient had a computed tomography with IV contrast, dialysis initiation in hospital, and outcome of the patients in Table 2. In our case series, there were two men and two women: 1 Caucasian and 3 African Americans. All of them developed acute kidney injury and mortality was 50%. We summarized creatinine trends during hospital stay in Table 3.

SARS-CoV-2 utilizes ACE2 and type 2 transmembrane serine proteases (TMPRSS 2) to enter the human cell [2]. The spike (S) protein of the virus binds to the catalytic domain of the ACE2 with high affinity [3], which can cause conformational changes in the S protein [4]. These cellular proteases (TMPRSS 2) are needed for the S protein priming, cleavage, and allow the fusion of viral and cellular membranes [2]. A prior study by Kuba and colleagues showed the first genetic proof that SARS-CoV needs ACE2 as a receptor in vivo [5]. Wrapp et al. have shown that on the cryoelectron microscopy, the S protein of the nCoV has 10- to 20-fold higher affinities to ACE2 compared to SARS-CoV [4].

Angiotensin-converting enzyme 2 (ACE2) is a zinc metalloprotease discovered in 2000 and has substantial homology with human angiotensin-converting enzymes (ACEs) [6]. The mRNA (messenger ribonucleic acid) of ACE2 is widely distributed in all human tissues, and the protein is widely expressed in the oral and nasal mucosa, nasopharynx, lung, gastrointestinal tract, kidney, and lymphoid tissue [7]. There is a high expression of ACE2 receptors on the epithelial cells of oral mucosa that plays a vital role in the transmission of COVID-19 [8]. Type II alveolar cells and the capillary endothelium express ACE2 receptors [7].

ACE2 is expressed in the proximal tubular cells significantly compared to other components in the kidney [9]. In kidney tubules, there is significant ACE2 expression on brush border and less expression in the cytoplasm [9]. The gene expression for ACE2 is 100-fold higher in kidney tissue compared to the lung [10]. There are significant proteinuria and hematuria in the patients infected with COVID-19 [10]. Of the patients who developed severe disease, a significant proportion of patients developed acute kidney injury and was associated with mortality [11].

Hypertension, diabetes, and cardiovascular disease are the common comorbidities associated with COVID-19 [11]. ACEI and ARB are the commonly used medications for the treatment of hypertension and kidney and heart diseases. Angiotensinogen is converted to angiotensin I by renin, which in turn is converted to angiotensin II (Ang II) by ACE. Angiotensin II activates angiotensin receptor type 1 (AT1R) and aldosterone and exerts vasoconstriction and sodium retention, resulting in hypertension. ACE2 converts angiotensin II to angiotensin (Ang)-(1–7), which exerts vasodilatory effects through the MAS receptor and thus negatively regulates the RAS system [12].

Angiotensin II causes inflammation, fibrosis, and edema through AT1R, and the absence of ACE2 causes the unrestricted activity of Ang II, which results in acute severe respiratory distress syndrome (ARDS). ACE2/Ang-(1–7)/Mas axis has an inhibitory effect on the signaling pathways of inflammation, fibrinogenesis, and cellular proliferation [12]. The net effect of ACE2 is anti-inflammatory, antifibrogenic, and antiproliferative [12]. A mice study showed that higher Ang II levels cause ACE2 to be internalized in the cell and undergo lysosomal degradation [13]. The internalization was prevented by losartan [13]. There is also speculation that the ACE2 and AT1R physically interact and form complexes on the cell membrane in the absence of Ang II, thus reducing the potential virus interaction with ACE2 [14].

Increased cardiac ACE2 gene expression and cardiac ACE2 activity were seen in Lewis rats after selective use of lisinopril or losartan, whereas the combination resulted in elevated cardiac ACE2 but not cardiac ACE2 mRNA [15]. In adult Sprague-Dawley rats, enalapril abated/mitigated the downregulation of ACE2 in the late phase of ventricular dysfunction of experimental myocardial infarction [16]. There was no increased cardiac ACE2 expression with ramipril or valsartan or in combination in the rat myocardial infarction model [17].

In a study of 79 patients that evaluated plasma ACE2 activity and long-term cardiovascular outcomes in patients with obstructive coronary artery disease (CAD), the ACE2 levels did not vary with age and the use of ACE1/ARBs did not alter the levels [18]. This study revealed that there were 83% of male patients in the high ACE2 level group [18]. In a study of 617 hypertensive patients that evaluated the urinary ACE2 level, only patients treated with olmesartan had increased levels [19]. The urinary ACE2 measured is soluble and is not membrane-bound [19].

Kuba et al., in a mouse model, showed that the RAS blockade attenuated the lung injury in SARS-CoV [5]. In a retrospective study of 1055 adult patients admitted with pneumonia, continued use of ACEI and statins resulted in lower rates of deaths and intubations [20]. Severe acute lung injury induced by influenza (H7N9) virus was observed in an experimental ACE2 knockout (KO) mouse model [21]. This study shows that ACE2 plays a critical role in pathogenesis; the blockade of AT1R resulted in the mitigation of acute lung injury [21].

Fang et al. hypothesized that the use of ACE2 modulation medications in hypertensive and diabetic patients increased the risk of developing severe COVID-19 infection [1]. This hypothesis has created widespread confusion in healthcare providers and was perpetuated by media channels and social media [22]. Many international societies have released statements on the continued use of the ACEIs/ARBs [14].

The understanding of COVID-19 and its pathogenesis sheds light on the potential targets for the treatment: development of spike subunit 1 protein-based vaccine, inhibiting viral spike protein priming proteases, through the serine protease inhibitor camostat mesylate, blocking ACE2 receptors to inhibit the viral interaction with ACE2 [23]; soluble ACE2 utilization to overwhelm the body membrane-bound ACE2 that can prevent the virus entry into the cell [24]; and baricitinib, which inhibits the endocytosis via G-associated kinase and AP2-associated protein kinase (AAK1), which is critical for virus entry [25].

There are currently few clinical trials underway to analyze ACE2 and RAAS blockade, a potential target for COVID-19: recombinant human angiotensin-converting enzyme 2 (rhACE2) as a treatment for patients with COVID-19 (NCT04287686) [26], losartan for patients with COVID-19 not requiring hospitalization (NCT04311177) [27], and losartan for patients with COVID-19 requiring hospitalization (NCT04312009) [27].

### 2.1. COVID-19 Infection and AKI

The incidence of AKI in patients infected with COVID-19 is around 3–15% as per recently published studies from China [11, 28]. The rates of AKI increased significantly to 14.5–50% in patients with severe COVID-19 infection requiring ICU admission [11, 29]. AKI in COVID-19 infection could be due to combination of cytokine-induced systemic inflammatory response and virus-induced direct cytotropic effect and acute tubular necrosis (ATN) due to multiorgan failure and shock. Other factors like volume depletion, drug toxicity, contrast exposure, and hypotension can result in AKI.

### 2.2. Acute Kidney Injury and Mortality with COVID-19 Infection

AKI is associated with increased mortality in patients infected with COVID-19. The incidence of AKI was 23% in 101 patients reported by Shi et al., who died from COVID-19 infection [30]. Elevated BUN, elevated baseline serum creatinine, peak serum creatinine >1.5, AKI stages 2 and 3, proteinuria, and hematuria are all associated with mortality as per Cheng et al., after adjusting for confounding factors [28]. Since AKI was associated with increased in-hospital mortality, the patients should be managed by providing hemodynamic support, avoiding nonsteroidal anti-inflammatory drugs (NSAIDs), nephrotoxins, and contrast along with early institution of continuous renal replacement therapy (CRRT).

## 3. Conclusion

In the above case series, all the patients developed acute kidney injury, but other factors were contributing to acute kidney injury like diuretics, iodinated contrast administration, hemodynamic instability apart from ACE inhibitors, and angiotensin receptor blockers. The ACE inhibitors and ARBs were stopped in these patients due to acute kidney injury.

There is no established evidence that suggests that the use of ACEIs/ARBs is associated with the COVID-19 or can potentiate the severe infection. Preclinical and clinical studies have shown beneficial evidence in viral pneumonia. The patients must follow the healthcare provider's advice before changing the medications. The medical professionals should follow the guidance of the national and international societies.

## Figures and Tables

**Table 1:** Laboratory data for all the patients on admission.

Patient	Sodium (mmol/l)	Potassium (mmol/l)	Bicarbonate (mmol/dl)	Blood urea nitrogen (mg/dl)	Serum creatinine (mg/dl)	CPK (units/L)	White cell count (K/mm^3^)	Lymphocyte count (K/mm^3^)	Urine analysis	Urine protein creatinine ratio (mg/g)
1	130	4.4	22	14	0.96	233	5.3	0.8	2+ protein and no RBC casts	Not available
2	130	4.0	34	22	0.87	458	6.9	0.6	No protein and no RBC casts	Not available
3	143	3.5	17	26	1.1	287	8.8	0.7	No protein and no RBC casts	524
4	154	3.2	23	25	1.8	278	10.6	0.3	3+ protein and no RBC casts (RBC—30/HPF)	270

**Table 2:** Clinical characteristics and outcome of the patients.

Patient	Age	Sex	Symptoms	Smoking	Comorbidities	ACEI or ARB	Diuretic use at home	Diuretic use in the hospital	Dialysis	Computed tomography with IV contrast	Outcome
1	49	Caucasian male	Fever, cough, SOB, chills, and body aches	Ex-smoker, 28-pack-year smoking history	Hypertension, diabetes, and dyslipidemia	Lisinopril 40 mg	HCTZ 25 mg	No	Yes	Yes	Died (DNR, comfort measures)
2	48	African American female	Dry cough and fever	Nonsmoker	Hypertension and dyslipidemia	Losartan 100 mg	HCTZ 25 mg	Yes	No	No	DNR, still remains critically ill and intubated
3	64	African American female	Cough, SOB, and fever	Nonsmoker	Hypertension, diabetes, and dyslipidemia	Azilsartan 80 mg	Spironolactone 25 mg	Yes	Yes	Yes	Died (patient made DNR)
4	77	African American male	Cough, SOB, and fever	Nonsmoker	Hypertension, dyslipidemia, and diabetes	Olmesartan 40 mg	No	No	No	No	Died (patient made DNR)

**Table 3:** Summary of serum creatinine trend during hospital stay.

Patient	Serum creatinine on presentation (mg/dl)	Serum creatinine values during hospital stay (mg/dl)	Serum creatinine level at outcome (mg/dl)
1	0.96	0.96 ⟶ 2.2 ⟶ 3.8 ⟶ 4.9	5.4
2	0.87	0.87 ⟶ 1.2 ⟶ 1.9 ⟶ 2.7	3.65
3	1.1	1.7 ⟶ 2.5 ⟶ 3.3 ⟶ 5.3	6.9
4	1.8	1.8 ⟶ 2.4 ⟶ 3.9 ⟶ 5.6	7.4

## Data Availability

The data used to support the findings of this study are available from the corresponding author on request.
